# Metformin Promotes Anti-tumor Biomarkers in Human Endometrial Cancer Cells

**DOI:** 10.1007/s43032-019-00019-2

**Published:** 2020-01-01

**Authors:** John Mark P Pabona, Alexander F Burnett, Dustin M Brown, Charles M Quick, Frank A Simmen, Maria Theresa E Montales, Shi J Liu, Tyler Rose, Iad Alhallak, Eric R Siegel, Rosalia CM Simmen

**Affiliations:** 1 Department of Physiology & Biophysics, University of Arkansas for Medical Sciences, Little Rock, AR USA; 2 Department of Obstetrics & Gynecology, Division of Gynecologic Oncology, University of Arkansas for Medical Sciences, Little Rock, AR 72205 USA; 3 The Winthrop P Rockefeller Cancer Institute, University of Arkansas for Medical Sciences, Little Rock, AR USA; 4 Department of Pathology, University of Arkansas for Medical Sciences, Little Rock, AR USA; 5 Department of Pharmaceutical Sciences, University of Arkansas for Medical Sciences, Little Rock, AR USA; 6 Department of Biostatistics, University of Arkansas for Medical Sciences, Little Rock, AR USA

**Keywords:** Metformin, Endometrial cancer, Estrogen receptor, Progesterone receptor, KLF9

## Abstract

Metformin (MET) is increasingly implicated in reducing the incidence of multiple cancer types in patients with diabetes. However, similar effects of MET in non-diabetic women with endometrial cancer (EC) remain unknown. In a pilot study, obese non-diabetic women diagnosed with type 1, grade 1/2 EC, and consenting to participate were randomly assigned to receive MET or no MET (control (CON)) during the pre-surgical window between diagnosis and hysterectomy. Endometrial tumors obtained at surgery (MET, *n* = 4; CON, *n* = 4) were analyzed for proliferation (Ki67), apoptosis (TUNEL), and nuclear expression of ERα, PGR, PTEN, and KLF9 proteins in tumor glandular epithelial (GE) and stromal (ST) cells. The percentages of immunopositive cells for PGR and for KLF9 in GE and for PTEN in ST were higher while those for ERα in GE but not ST were lower, in tumors of MET vs. CON patients. The numbers of Ki67- and TUNEL-positive cells in tumor GE and ST did not differ between groups. In human Ishikawa endometrial cancer cells, MET treatment (60 μM) decreased cell numbers and elicited distinct temporal changes in *ESR1*, *KLF9*, *PGR*, *PGR-B*, *KLF4*, *DKK1*, and other tumor biomarker mRNA levels. In the context of reduced *KLF9* expression (by *siRNA* targeting), MET rapidly amplified *PGR*, *PGR-B*, and *KLF4* transcript levels. Our findings suggest that MET acts directly in EC cells to modify steroid receptor expression and signaling network and may constitute a preventative strategy against EC in high-risk non-diabetic women.

**Electronic supplementary material:**

The online version of this article (10.1007/s43032-019-00019-2) contains supplementary material, which is available to authorized users.

## Introduction

Endometrial cancer (EC) is the most common gynecologic malignancy in the USA, with approximately 60,000 new diagnosed cases per year [1]. Obesity is a major risk factor in the development of EC [2, 3], conferring a 1.6-fold increase in disease risk for every 5 kg/m^2^ increase in body mass index (BMI). Obesity is also associated with insulin resistance and an increased risk (1.5–2-fold) in developing type 2 diabetes (heretofore referred as diabetes) [4, 5]. Given the rising obesity rates in the female population and that 1 in 7 adult women are diagnosed with diabetes [6], it is anticipated that EC cases will exponentially rise in the imminent future, invoking a critical need for new treatments and efficacious preventative strategies to impede its development and progression.

Metformin (1,1-dimethylbiguanide hydrochloride; MET) is an oral hypoglycemic agent used in the management of diabetes. MET lowers blood glucose levels by inhibiting gluconeogenesis and improves insulin sensitivity by increasing peripheral glucose uptake and utilization [7, 8]. The use of MET in diabetic patients is associated with significantly lower risks of cancer incidence and mortality [9–12]. More germane to this study, EC patients with diabetes using MET exhibited improved overall and progression-free survival [11, 13, 14]. Whereas the therapeutic benefits of MET in many cancer types are known to be mediated by the inhibition of the PI3K/AKT/mTOR-signaling pathways [7, 15], the anti-cancer effects and signaling mechanisms of MET in non-diabetic vs. diabetic women with EC have not been fully characterized. In one recent study, pre-surgical MET treatment of EC patients without diabetes was associated with reductions in levels of tumor Ki67 and of tumor mTOR-associated phosphoproteins [16]. However, similar studies to confirm these findings are limited. Understanding the full complement of the underlying network(s) associated with MET effects in non-diabetic women with EC is necessary to expand the therapeutic benefits of MET in this patient population.

The present study examined the short-term effects of MET on tissue proliferation, apoptosis, and expression of estrogen receptor-α (ERα), progesterone receptor (PGR), specificity protein (Sp)–related transcription factor Krüppel-like factor 9 (KLF9) and tumor suppressor phosphatase and tensin homolog (PTEN) in endometrial tumors of non-diabetic obese women with EC. The tumor biomarkers identified to be MET-responsive in vivo were further evaluated for MET effects in vitro using the human endometrial cancer cell line Ishikawa. The collective results identify novel networks related to direct MET effects on endometrial carcinoma cells.

## Materials and Methods

### Human Samples

**Fig. 1:**
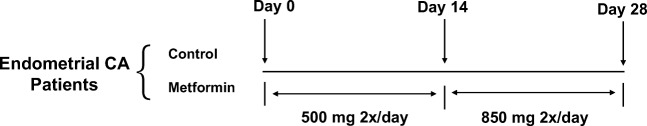
Treatment schedule. Non-diabetic obese women diagnosed with endometrial cancer (CA) were consented to the study (Clinical Trial No. NCTO1877564) and randomized to receive no metformin or metformin within a week of diagnosis. Metformin was orally administered 2× per day, with patients receiving a lesser dose in the first 2 weeks and a higher dose in the last 2 weeks of the study. Patients underwent hysterectomy 28 to 32 days after initial diagnosis and excised tumors were used for further analyses as described under “Materials and Methods”

Human subject protocols were approved by the University of Arkansas for Medical Sciences Institutional Review Board. Patient recruitment was listed under Clinical Trial No. NCTO1877564. Eligibility criteria were non-diabetic obese women (BMI ≥ 30) with histologically confirmed EC and no diagnosis of other cancers, who show normal liver and renal function, and who consented to the study. Exclusion criteria were smoking, previous metformin use, or regular alcohol consumption. A total of 13 patients were enrolled within a period of 1 year (2013–2014) and were randomized to receive either oral MET or no MET for 28–32 days during the pre-surgical window between diagnosis and hysterectomy (Fig. 1). The dose-escalation schedule of 500 mg oral MET twice a day for 14 days, followed by 850 mg twice a day for 14–18 days, followed currently used therapeutic doses recommended for preventing gastrointestinal side effects [17]. Blood samples were collected for all participants on day 1 (prior to start of MET for subjects randomized to treatment) and were analyzed for fasting blood glucose and other clinical parameters. Patients were provided MET pills for free to encourage compliance and were monitored for potential adverse effects of MET intake by telephone. At the end of the study period, hysterectomy was performed and excised tumors were histologically classified and graded by our collaborating pathologist (CMQ).

### Immunohistochemistry

Tumor samples were formalin-fixed and paraffin-embedded; sections (5 μm) were processed by heat-induced epitope retrieval (citrate buffer) and subsequent incubation with designated antibodies as previously described [18]. Table 1 presents the primary antibodies (Research Resource Identifier (RRID), antibodyregistry.org) and their working dilutions used at incubation conditions of 4 °C for 16–24 h. Immunoreactivity was detected using the VECTASTAIN Elite ABC Kit (Vector Laboratories) and biotinylated anti-rabbit secondary antibodies (Vector Laboratories), and slides were counterstained with hematoxylin. The stained sections were digitized using the Leica Digital Pathology Whole Slide Scanner (Aperio ImageScope). The number of nuclear-stained and non-stained glandular epithelial (GE) and stromal (ST) cells in 3–4 random visual fields (~ 100 cells per field) for each tissue section was separately counted manually. Cells were scored as non-staining (i.e., background staining) based on sections that were processed in parallel with the omission of the primary antibody. Data are expressed as a percentage of the number of nuclear-stained cells, relative to the total number of cells (stained + non-stained) counted.

**Table 1:** Antibodies used in the study

Protein	Vendor/catalog no.	RRID^a^	Working dilution
Estrogen receptor α	Santa Cruz/sc542	AB_63140 (IHC)^b^	1:250
Ki67	Abcam/Ab16667	AB_302459 (IHC)^b^	1:200
Krüppel-like factor 9	Lifespan/LSB5581	AB_10912289 (IHC)^b^	1:200
Progesterone receptor	Santa Cruz/sc7208	AB_2164331 (IHC)^b^	1:200
Phosphatase and tensin homolog	Cell Signaling/138G6	AB_823618 (IHC)^b^	1:200
Krüppel-like factor 9	Abcam/Ab124145	AB_10972187 (WB)^c^	1:1000
Lamin A	Abcam/Ab26300	AB_775965 (WB)^c^	1:1000

^a^Research Resource Identifier (antibodyregistry.org)

^b^IHC used for immunochemistry

^c^WB used for Western blot

### Metformin Treatments In Vitro

The human endometrial epithelial carcinoma cell line Ishikawa (a gift of Dr. Bruce Lessey, Greenville Health System) was previously authenticated and determined to express functional estrogen and progesterone receptors [19]. Cells were grown and propagated in Minimal Essential Media (MEM) containing phenol red and supplemented with 10% (v/v) fetal bovine serum (designated MEM-FBS-phenol red) and 1% (v/v) antibiotic-antimycotic solution (Gibco), in a humidified incubator (5% CO_2_/95% air) at 37 °C. Metformin (MET; Sigma-Aldrich) was dissolved in phosphate-buffered saline (PBS; Gibco) and used at a final concentration of 60 μM for treatments. This dose approximates the plasma concentration of MET in diabetic patients treated with the drug [20]. Cell viability assay was carried out in 96-well culture plates (Corning Ware), with each well initially seeded with 5 × 10^3^ cells in MEM-FBS (10%, v/v)-phenol red. Cells were synchronized by serum starvation in MEM-phenol red containing 2% FBS (v/v) for 24 h and then transferred to MEM-FBS (10%)-phenol red for treatment with MET or vehicle control (PBS). Cellular apoptosis was measured using cells initially plated in 12-cm dishes, and 24 h later, cells were treated with MET. For gene expression analysis, cells were seeded at a density of 1 × 10^5^ per well in 12-well plates and cultured to 50–60% confluence in MEM-FBS-phenol red prior to MET treatments. For the 48-h incubation period, cells were replenished with fresh media containing MET or no MET at 24 h.

### Viability, Apoptosis, and Gene Expression Assays

Treated cells were collected at select time points for analyses. Cellular viability was evaluated at 48 h post-initial treatment (MET or PBS) by trypan blue exclusion using the Vi-Cell cell viability analyzer (Beckman Coulter Inc.). MET- or PBS-treated cells were stained with annexin V and propidium iodide (Trevigen) at 48 h post-initial treatment and quantified for percent of apoptotic cells in the early (annexin V–positive/propidium iodide–negative) and late (positive for both annexin V and propidium iodide) stages using the Becton Dickinson LSRFortessa Flow Cytometer, as described previously [21]. RNA isolation, preparation of cDNAs, and QPCR analyses were performed following published studies [22]. Primers (Supplemental Table S1) were designed to span introns and synthesized by Integrated DNA Technologies. mRNA expression was calibrated to a standard curve generated using pooled cDNA stocks, and TATA-box binding protein (*TBP*) mRNA was used as the normalizing control.

### Transient Transfections with siRNAs

Ishikawa cells, at 60–70% density, were transiently transfected with non-targeting (scrambled control, scr; 50 nM concentration) small interfering RNAs (siRNAs) and siRNAs directed against *KLF9* (50 nM concentration) (Dharmacon) using Lipofectamine 2000 (Invitrogen), as previously described [23]. Cells were treated with MET to a final concentration of 60 μM for 2 h and processed for gene expression analysis as described above. Nuclear extracts prepared from MET-treated cells transiently transfected with scr or *KLF9* siRNAs were processed for Western blot analyses using rabbit anti-rat KLF9 antibody (Table 1), following previous studies [23]. Blots were stripped and re-probed with antibody against Lamin A, which was used as the normalizing control for protein loading.

### Data Analysis

Statistical analyses were performed using SigmaStat (version 3.5; Systat Software). Data (mean ± SEM) were analyzed for statistical significance (*P* ≤ 0.05) by one-way ANOVA followed by Tukey’s post hoc test (for three or greater groups) or by Student’s *t* test (for two groups).

## Results

### Demographic Information for Study Population

A total of 13 women were enrolled in the study, 6 of whom received MET and 7 of whom received no drug during the preoperative window between diagnosis and hysterectomy. Of these, 5 were subsequently determined to fail the established criteria (e.g., found to be a regular smoker), were lost to follow-up, and/or did not complete the MET treatment regimen. Four patients from each group who successfully completed the study provided the tumor samples used in the analyses. The two groups were comparable in age and BMI and showed normal fasting blood glucose levels, the latter confirming their non-diabetes status (Table 2). Other measured clinical parameters also did not differ between the two groups (Table 2). Histological pathology indicated that all women in the study had type 1 (grade 1/2) EC.

**Table 2:** Patient demographics

Parameters^a^	Control (*n* = 4)	Metformin (*n* = 4)	*P* value
Age (years)	60.5 ± 1.8	55.4 ± 4.7	0.36
BMI (kg/m^2^)	38.2 ± 2.8	42.5 ± 4.9	0.49
Fasting blood sugar (mg/dL)	93.7 ± 6.4	92.8 ± 5.1	0.91
Hemoglobin (g/dL)	10.7 ± 1.9	12.6 ± 0.5	0.19
White blood cells (× 10^3^/μL)	7.0 ± 0.4	7.3 ± 1.2	0.42
Calcium (mg/dL)	9.1 ± 0.2	8.8 ± 0.3	0.25
Creatinine (mg/dL)	0.78 ± 0.05	0.80 ± 0.04	0.35

^a^Data were collected from patients prior to the start of the treatment regimen

### Effects of Metformin on Tumor Biomarkers

**Fig. 2:**
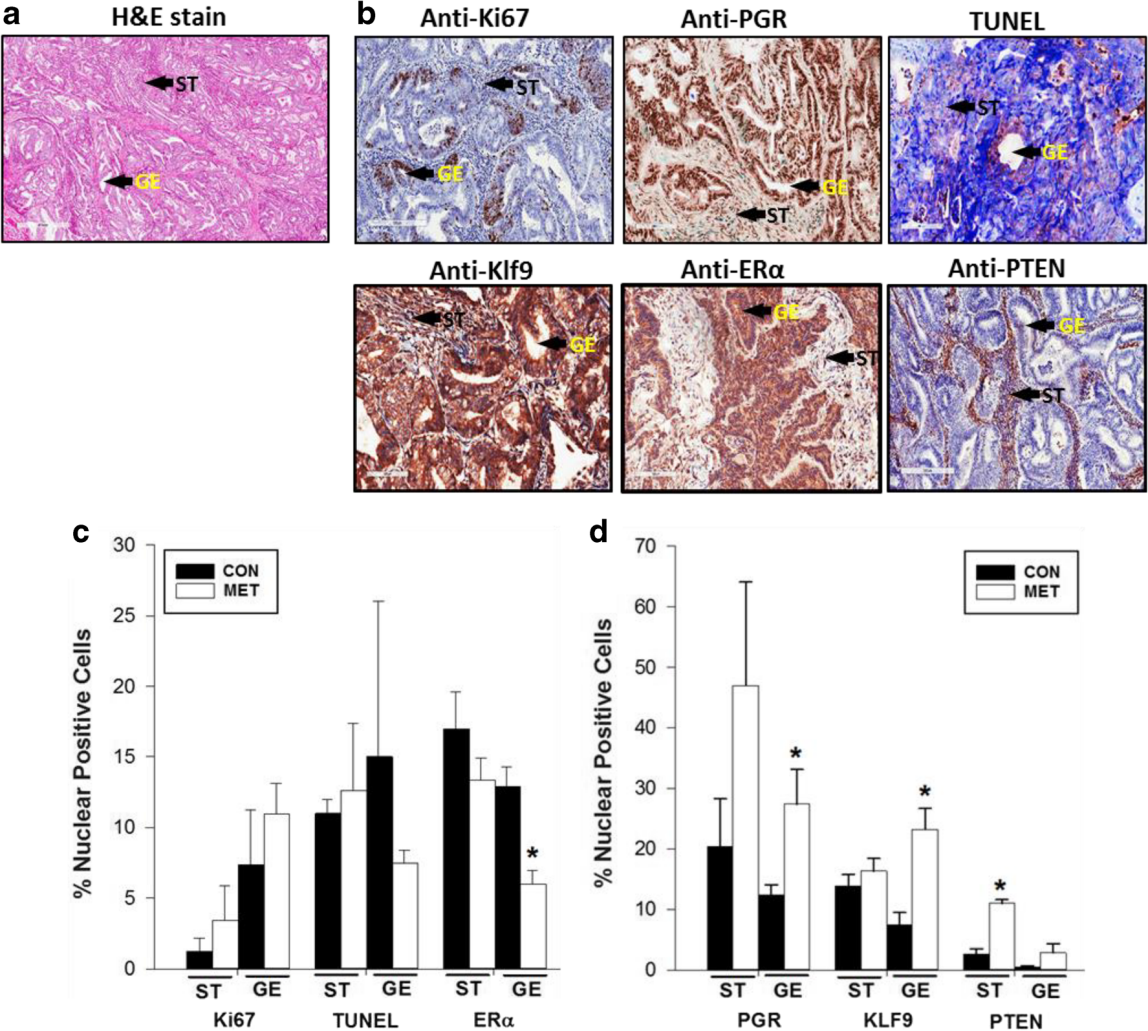
Expression of tumor-associated proteins in non-diabetic EC patients with or without metformin treatment. **a** Representative H&E-stained section of tumor tissue from a non-MET patient undergoing hysterectomy after EC diagnosis. **b** Tumor sections were processed for immunohistochemistry as described under “Materials and Methods” using previously characterized antibodies (Table 1)*.* Immunopositive tumor stromal (ST) and glandular epithelial (GE) cells were identified as brown staining. Arrowheads refer to ST and GE. **c**, **d** The percentages of nuclear-localized Ki67, TUNEL, and ERα (**c**) and PGR, KLF9, and PTEN (**d**) in ST and GE cells of tumor tissues were determined by counting the number of positive-staining nuclei over the total number of cells counted per field. Data (mean ± SEM) represent analyses of tissue sections from *n* = 4 patients per group (CON or MET). For each tissue section, 3–4 random visual fields were counted. *P < 0.05 by Student’s paired *t* test between CON and MET groups for each cell type. Bars without asterisks indicate no significant difference between CON and MET groups

Immunohistochemistry was used on patient tumor samples to assess the relative expression of proteins that are known to be positively (Ki67; estrogen receptor-α (ERα)) and negatively (progesterone receptor (PGR); Krüppel-like factor 9 (KLF9); phosphatase and tensin homolog (PTEN)) associated with EC. A representative H&E-stained section of tumor tissue (Fig. 2a) and representative immunostained slides of hysterectomy specimens with different antibodies (Fig. 2b) are shown. Immunoreactive proteins identified by respective antibodies were located in the nuclear and/or cytoplasmic compartments of tumor stromal (ST) and glandular epithelial (GE) cells. The expression levels of nuclear-localized proteins were quantified and found to differ with cell type in response to MET (Fig. 2c, d). The percentages of nuclear-positive Ki67 cells in both tumor ST and GE were comparable between MET-treated and untreated groups (Fig. 2c). The level of apoptosis, evaluated by TUNEL assay, was also not significantly affected for ST and GE with MET treatment (Fig. 2c). A similar lack of MET effect relative to control was found for immunoreactive ERα, PGR, and KLF9 in ST (Fig. 2c, d). However, for the corresponding GE, MET reduced the percentage of nuclear-positive cells for ERα (by 2-fold) and increased the percentage of nuclear-positive cells for PGR (by 2-fold) and for KLF9 (by 2.5-fold), respectively (Fig. 2c, d). Basal (untreated group) PTEN expression was more pronounced in ST than in GE (Fig. 2b); MET robustly increased nuclear PTEN levels (by 4-fold) in ST and elicited a numerical albeit non-significant increase in GE (Fig. 2d).

### In Vitro Effects of Metformin

**Fig. 3:**
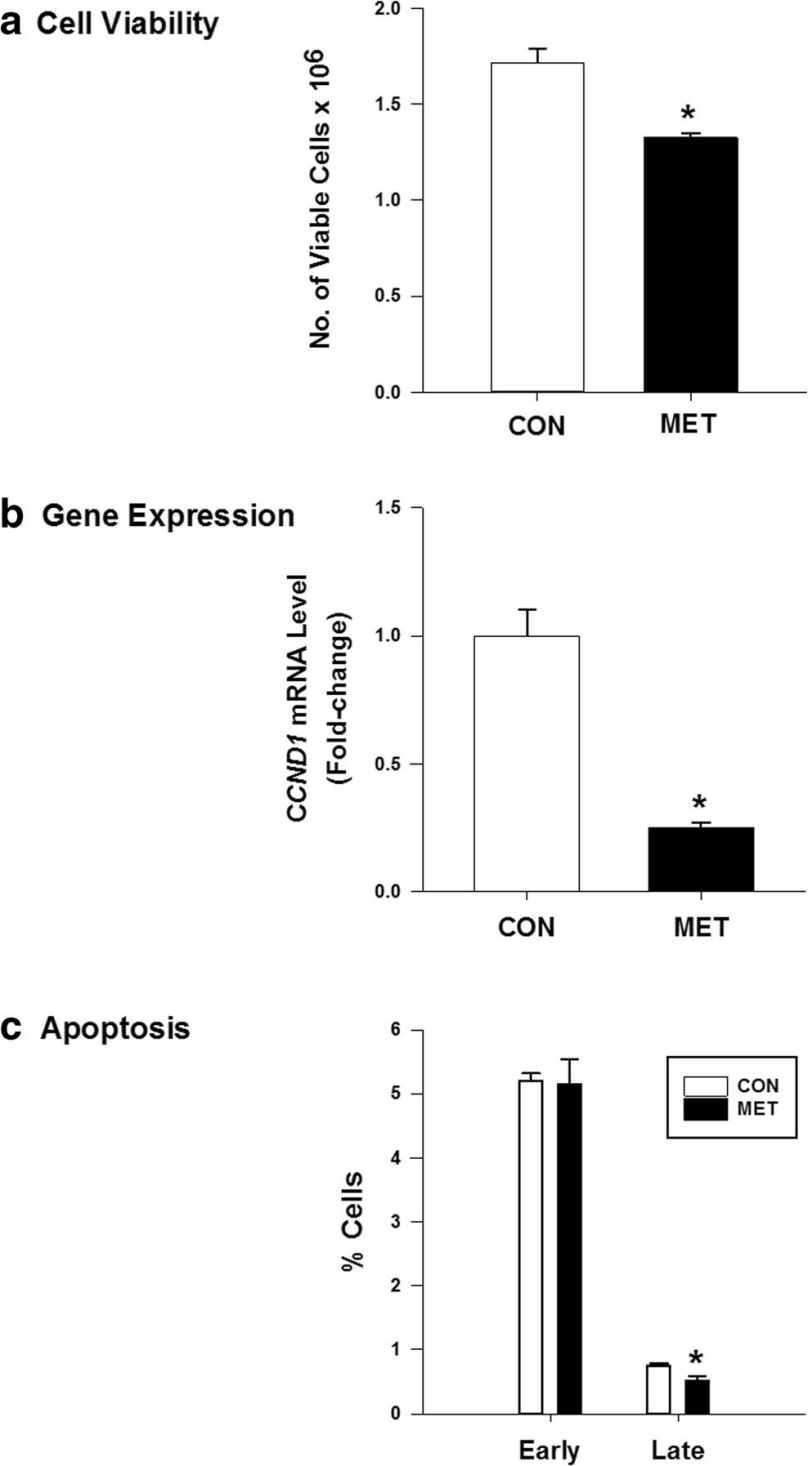
Metformin effects on human Ishikawa carcinoma cell viability and apoptotic status. Cells were treated without (CON) or with MET (60 μM) and evaluated for **a** cell viability, **b***CCND1* mRNA levels, and **c** apoptotic status, as described under “Materials and Methods.” TATA-binding protein mRNA was used as the normalization control for *CCND1* mRNA levels in **b**. Data (mean ± SEM) are from three independent experiments. *P < 0.05 by Student’s paired *t* test between CON and MET groups

To determine whether the observed effects of short-term MET on tumor samples resulted from its direct actions on tumor epithelial cells, the human Ishikawa cell line, which was derived from the epithelial component of a well-differentiated endometrial adenocarcinoma [24], was treated with MET, and the parameters measured in vivo (gene expression, proliferation, apoptosis) were similarly evaluated in vitro. Relative to control (PBS) cells, MET decreased the number of viable cells (Fig. 3a) and reduced cyclin D1 (*CCND1*) mRNA levels (Fig. 3b) 48 h post-treatment. MET had no effect on the proportion of apoptotic cells in the early stage (Fig. 3c). The percentage of apoptotic cells in the late stage was very low (< 1%), but MET showed a modest but significant effect (Fig. 3c).

**Fig. 4:**
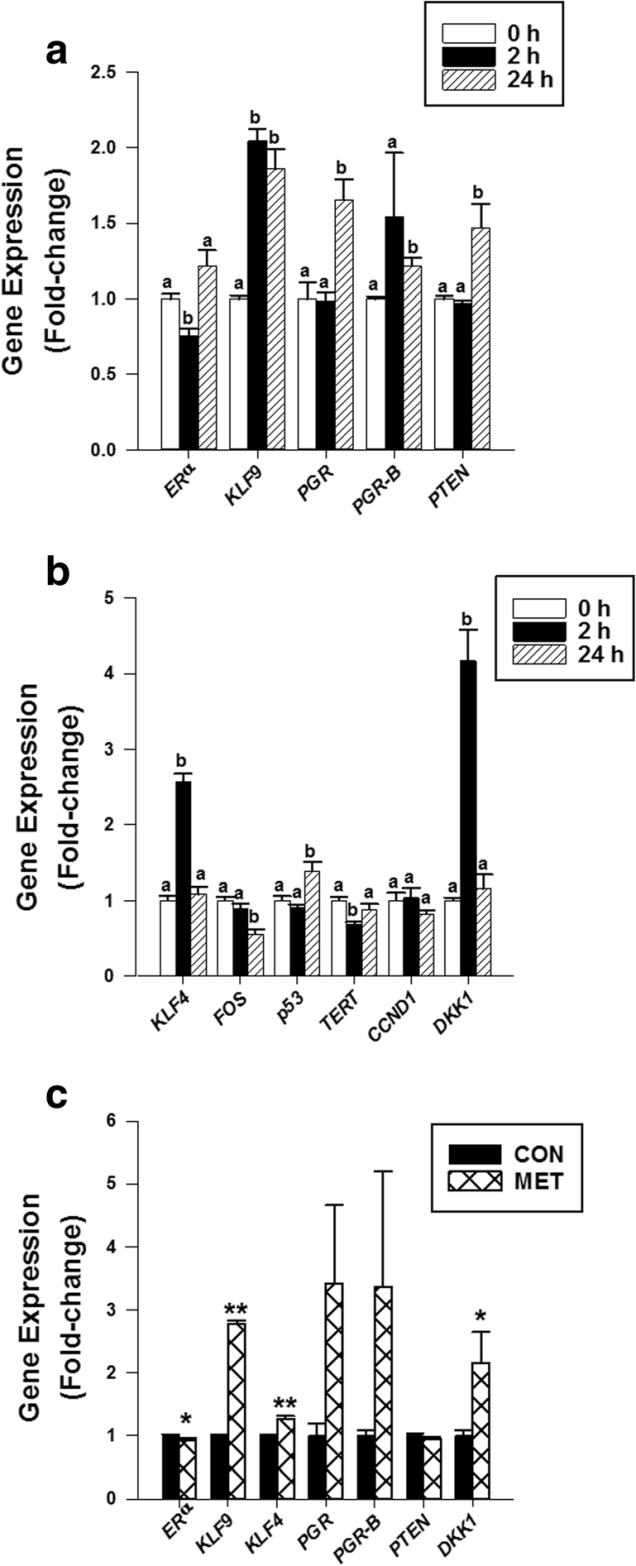
Metformin treatment time-dependently modified gene expression in human Ishikawa carcinoma cells. **a**, **b** Cells were treated with MET (60 μM) for 2 h and 24 h and evaluated for specific mRNA levels by QPCR. Cells treated with MET and immediately collected (time 0) served as controls. **c** Cells treated with PBS (CON) or MET (60 μM) 2× (at 0 h and 24 h) were collected 24 h after the last treatment (at 48 h) and evaluated for specific mRNA levels by QPCR. TATA-binding protein mRNA was used as the normalization control for all RNA transcripts. Data (mean ± SEM) are expressed as fold change from corresponding controls and were obtained from three independent experiments. **a**, **b** For each gene, means among bars showing different superscripts differed at *P < 0.05, as determined by one-way ANOVA, followed by Tukey’s test. For each gene in **c**, means for CON and MET groups were determined for significant differences by Student’s paired *t* test. **P < 0.001; *P < 0.05

Gene expression in Ishikawa cells was affected by MET in a time-dependent manner (Fig. 4). Relative to *t* = 0 (control), *ERα* mRNA levels were reduced early (at 2 h) by MET treatment, but this suppression was not sustained at 24 h post-treatment (Fig. 4a). An early effect at 2 h (2-fold increase), which persisted to 24 h, was noted for *KLF9* (Fig. 4a). By contrast, MET increased transcript levels for total *PGR* (1.8-fold), *PGR-B* isoform (1.25-fold), and *PTEN* (1.5-fold) transcript levels by 24 h but not earlier at 2 h (Fig. 4a).

The expression of other genes that are implicated in EC, owing to their tumor-promoting or tumor-repressive functions, was evaluated in MET-treated Ishikawa cells at the same time points (2 h and 24 h post-MET treatment). The analyses for the same genes were not performed in EC tumors due to limited tissue amounts. Tumor suppressors Krüppel-like factor 4 (*KLF4*; at 2 h) and *p53* (at 24 h) transcript levels were elevated, while those of tumor promoters *c-FOS* (at 24 h) and telomerase (*TERT*; at 2 h) were reduced (relative to *t* = 0), with MET treatment (Fig. 4b). *CCND1* transcript levels did not change with MET at 2 h and 24 h. The transcript abundance of Dickkopf-related protein 1 (DKK1), a well-known progesterone-regulated gene [25], was markedly elevated (by 4-fold) within 2 h of MET treatment and returned to basal levels at 24 h (Fig. 4b).

The transcript levels of a select number of genes were evaluated for response to MET after an extended treatment period (post-48 h; 2× treatments at 0 and 24 h) (Fig. 4c). Relative to CON, MET-treated cells showed reductions in *ERα* mRNA levels, inductions in *KLF9*, *KLF4*, and *DKK1* mRNA levels, and no effect on *PTEN* mRNA levels. *PGR* (Total) and *PGR-B* mRNA levels were numerically higher with MET, but the increase did not reach the level of significance (*P* = 0.06).

### KLF9-Dependent Metformin Effects on Progesterone Receptor Expression

**Fig. 5:**
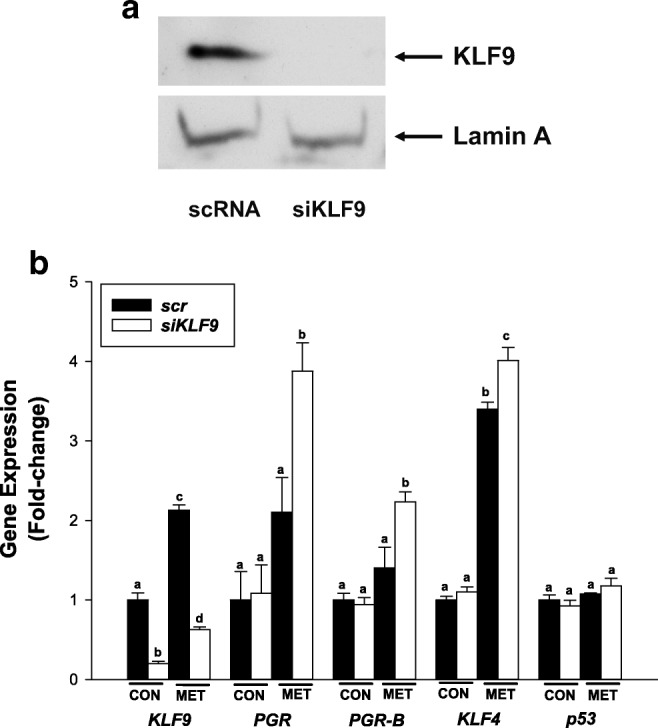
Metformin increased *PGR* and *KLF4* transcript levels in cells with reduced *KLF9* levels. **a** Human Ishikawa cells were transiently transfected with control (scrambled siRNAs, *scr*) or *KLF9* siRNAs as described under “Materials and Methods.” Nuclear extracts were prepared from transfected cells and analyzed by Western blots using antibodies against KLF9 and Lamin A (Table 1). Protein expression of KLF9 (top) and of Lamin A (bottom) are shown. **b** Cells transiently transfected with *scr* (control) siRNAs and *KLF9* siRNAs were incubated in media without and with added MET (60 μM). Cells were collected 2 h after treatments and quantified for transcript levels (*KLF9*, *PGR*, *PGR-B*, *KLF4*, and *p53*) by QPCR. Data (mean ± SEM; *n* = 3 independent experiments) were normalized to TATA-binding protein mRNA and are expressed as the fold-change relative to scr siRNA–transfected control cells. Significant differences among groups for each gene (*P* < 0.05) are designated with different superscripts and identified by one-way ANOVA, followed by Tukey’s test

*KLF4* is a PGR-regulated gene [26] and KLF9 is a PGR (specifically PGR-B)-interacting protein [27–29] in endometrial epithelial cells. We examined whether MET affects the functional linkage between KLF9, KLF4, and PGR in EC cells transiently transfected with siRNAs directed against KLF9 and in parallel with control (scr) siRNAs. To confirm siRNA transfection efficiency, control (non-MET treated) cells transfected with *KLF9* siRNAs or *scr* siRNAs were analyzed for KLF9 protein (by Western blot of nuclear lysates) and *KLF9* transcript levels (QPCR). The reduction in nuclear KLF9 protein (Fig. 5a) and transcript (Fig. 5b) levels in si*KLF9*, relative to *scr* siRNA–transfected cells, confirms the efficiency of *KLF9* siRNA targeting. The loss of KLF9 expression (transcript and protein levels) in *siKLF9*-transfected cells was accompanied by rapid and concurrent increases (within 2 h) of total *PGR*, *PGR-B* isoform, and *KLF4* transcript levels. The specificity of these effects was displayed by the absence of comparable changes elicited on *p53* mRNA levels (Fig. 5b).

## Discussion

The present study provides support for a role for MET in inhibiting EC. The in vivo (proof-of-concept) study involved a short-term MET treatment (pre-surgical; between diagnosis and hysterectomy) of non-diabetic obese women with EC, while the in vitro experiments analyzed the direct effects of MET on proliferation, apoptosis, and gene expression in the human endometrial carcinoma cell line Ishikawa. While the in vivo results were limited by the small patient sample size due in part to the prevalence of type 2 diabetes in obese women with EC [3], the non-diabetic obese control and treatment groups did not differ in BMI and age, thus precluding the confounding issues of insulin resistance, adiposity, and/or menopausal status found in other studies that had previously evaluated the effects of MET in EC [14, 30–34]. Importantly, the congruence of changes elicited by MET in vivo (protein) and in vitro (mRNA) on the expression of PGR, PTEN, KLF9, and ERα provides mechanistic underpinnings to the direct effects of MET on EC. Results implicate the preventative potential of MET in non-diabetic obese women who are at risk for EC through MET’s effects on steroid receptor signaling pathway(s).

Our primary endpoints for MET outcomes in vivo were proliferation (Ki67 immunostaining) and apoptotic (TUNEL assay) status and changes in nuclear levels of tumor biomarker proteins ERα, PGR, KLF9, and PTEN. Reported outcomes of MET on proliferation and apoptosis markers in tumors of patients with EC have been inconsistent, with some showing no 33 and others showing inhibitory [15, 31] effects. We found that MET had no effect on proliferation and apoptotic indices in the stromal and epithelial compartments of existing tumors (Fig. 2c), which may be a function of short-term MET exposure and/or the non-diabetic status of the patients. By contrast, we found that MET modified the nuclear levels of immunoreactive ERα, PGR, and KLF9 proteins in tumor tissues and preferentially affected GE relative to ST. The parallel increase in nuclear levels of PGR and KLF9 with MET in vivo is consistent with their demonstrated functional interactions in EC cells [28, 29]. Moreover, the corresponding decrease and increase in nuclear levels, respectively, of ERα and KLF9 with MET are congruent with the negative regulation of ERα expression by KLF9 in human EC cells [35]. Further, the lower expression of PTEN in tumor epithelial cells (~ 4× lower than in tumor stroma; Fig. 2d) concurs with PTEN loss in tumor epithelial cells as a diagnostic marker of endometrial pre-cancers [36] and with the linkage between loss of PTEN and activation of ERα signaling in EC [37, 38]. While it is not possible to directly compare the MET-elicited changes in epithelial ERα, PGR, and KLF9 proteins in vivo with those of the time-dependent changes in their respective transcripts in Ishikawa cells in vitro, it is noteworthy that the in vitro responses to MET were achieved at a concentration (60 μM) significantly less than previously reported (predominantly mM and hence pharmacological range) for most studies. We have also shown that MET at the same dose (60 μM) and treatment duration (24 h) increased *PTEN* mRNA levels in human colon cancer cells [21]. Moreover, the transcript levels of *KLF4*, *cFOS*, *p53*, and *TERT* were altered by MET in the direction consistent with their respective anti- (increased for *KLF4* and *p53*) and pro- (decreased for *cFOS* and *TERT*) tumorigenic activities. ERα is known to directly bind to p53 tumor suppressor to repress its function [39], and *TERT* expression is transcriptionally enhanced by the binding of ERα to *TERT* promoter [40]. Our results suggest that MET directly targets EC cells to modify the expression of a subset of tumor-associated genes, in part through mechanisms involving steroid receptor signaling.

ERα may constitute a key mediator of MET in targeting EC, but the underlying mechanism for this remains unknown. Similar to our findings reported here in non-diabetic obese women with EC, MET caused a reduction of ERα expression in endometrial tumors of diabetic women relative to non-treated women [32]. In diabetic patients with EC, preoperative MET significantly decreased insulin, insulin-like growth factor-1, and leptin [30, 33], all of which are positive regulators of ERα expression and/or activity [41–43]. In a study of pre-diabetic obese women with increased risk for EC, MET intake for 16 weeks produced no significant changes in serum IGF-I and insulin [44], suggesting that the changes in serum levels of these growth factors with MET may be a specific response of EC patients with full-blown diabetes. We were unable to evaluate serum hormone levels in our patient pool due to lack of patient plasma samples at the conclusion of the study. Nevertheless, our results showing that Ishikawa cells, when treated with MET under conditions of normal glucose (recapitulating non-diabetes status), displayed a parallel reduction in *ERα* transcript levels comparable with that noted for ERα protein with MET intake in vivo preclude the exclusive participation of systemic factors and implicate direct mechanisms in MET-elicited responses. Consistent with our results, cell lines established from endometrial tumor tissues obtained from women undergoing surgery for EC showed reduced *ERα* transcript levels with MET [45].

Ligand-activated PGR has been implicated as a tumor suppressor in EC [46]. We showed here that various components of the PGR signaling pathway were altered with MET treatment of Ishikawa cells, similar to our in vivo results. Nevertheless, while the increased levels of pathway effector (PGR, PGR-B), co-regulator (KLF9), and downstream gene targets (*KLF4*, *DKK1*) may suggest a simple linear pathway by which MET’s elevation of PGR expression can orchestrate anti-tumorigenic outcomes, this may not necessarily be the case, given the temporal course in gene expression changes. For example, the relative increases in *KLF9*, *KLF4*, and *DKK1* transcript levels (by 2 h) preceded those of *PGR* and *PGR-B*, which were observed at 24 h. Further, while the early increase in *KLF9* transcript levels was maintained through 24 h and 48 h post-MET, *DKK1* and *KLF4* transcript levels were reduced to basal levels by 24 h and needed additional MET treatment to rise to levels above controls (seen at 48 h; Fig. 4c). *ERα* transcript levels also showed a rapid response to MET (a decrease by 2 h), but this reduction was not observed by 24 h and similarly required additional MET treatment to be maintained below those of control cells. Taken together, our results suggest that MET may elicit early (*ERα*, *KLF9*, *KLF4*, and *DKK1)* and late (*PGR*, *PGR-B*, and *PTEN*) responses on PGR/ERα gene networks and that maintenance of some of these MET effects on gene expression (*ERα*, *KLF4*, *DKK1*, and *PGR)* may require additional/continuous exposure to MET, possibly involving the activation of other signaling pathways. In this regard, the recent report that the development of EC tumors in a xenograft mouse model was significantly suppressed by the combined administration of MET and medroxyprogesterone acetate, when compared to individual treatments [47], is consistent with this possibility. Moreover, induction of PGR expression in Ishikawa cells by MET, which was shown to be mediated by the activation of the AMPK pathway at pharmacological (mM) doses, was only partially inhibited by an AMPK inhibitor [48].

We previously reported the loss of KLF9 expression in EC tumors [23]. Although a direct mechanistic link between EC pathogenesis and KLF9 loss of expression has yet to be evaluated, our findings are consistent with the increasing validation of KLF9 tumor-suppressive functions in mouse models of cancer and in cancer cell lines [49–53]. Here, we showed that induction of KLF9 expression by MET in GE of EC tumors in vivo is recapitulated in Ishikawa cells in vitro; in the latter, the robust (early) increase in *KLF9* transcript levels with MET was temporally and closely aligned with, respectively, the reduction of *ERα* and *TERT* and the induction of *KLF4* and *DKK1* transcript levels and preceded the expression changes noted for *PGR*, *PGR-B*, *PTEN*, *cFOS*, and *p53* transcripts. Given that MET induction of *KLF9* transcript levels occurred early and was maintained at all time points examined (2 h, 24 h, and 48 h), we suggest that KLF9 is a major component of MET signaling. By siRNA targeting of *KLF9* in Ishikawa cells to mimic the low levels found in EC tumors [23], we found that MET may compensate for the progressive loss of *KLF9* in tumor cells by rapidly increasing transcript levels for *PGR*, *PGR-B*, and *KLF4*, all of which exhibit anti-tumor properties. The lack of a similar effect of *KLF9* siRNA targeting on *p53* transcript levels in the background of MET indicates the specificity of MET effects on KLF9-associated signaling.

**Fig. 6:**
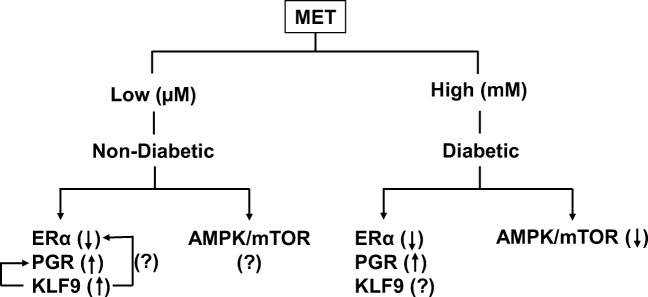
Proposed model of MET effects on EC cells. In non-diabetic women, low levels of MET (μM dose) may be sufficient to elicit specific effects on steroid receptor expression and signaling, whereas in women with diabetes, high levels (mM) of MET may result in, respectively, activation of steroid receptor and inhibition of metabolism-associated (AMPK/mTOR) signaling. Up- and down-directed arrows refer to up- and downregulation of gene/protein expression. Arrow between KLF9 and PGR indicates functional regulation of PGR by KLF9. (?) indicates current unknowns

The present study did not address how MET may directly act on EC cells to influence the expression of genes with pro- and anti-tumor effects. A well-established mechanism of MET action involves the inhibition of mammalian target of rapamycin (mTOR) signaling via increased phosphorylation and hence activation of AMPK. A recent study using high MET doses (5 mM) reported the inhibition by MET of *ERα* expression and estrogen-mediated proliferation of Ishikawa cells through AMPK activation and subsequent inhibition of mTOR signaling [54]. Reduction of phospho-AKT and phospho-p44/42 MAPK levels was also observed with MET treatment in these cells, suggesting MET’s influence on kinases that control expression of genes involved in growth regulation. How MET-mediated changes in the activity of kinases directly affect steroid receptor signaling in EC cells remains unclear. However, steroid receptor transcriptional activities are highly regulated by phosphorylation events [55, 56], and endocrine-related cancers are associated with aberrant phosphorylation of steroid receptors leading to inappropriate interaction with co-regulators [57, 58]. Based on our findings and those of others [45, 48, 54], we posit that MET may exhibit dose-dependent signaling pathways in EC (Fig. 6). At low MET (μM) concentrations in the context of normal glucose (non-diabetic) levels, steroid receptor expression and signaling involving KLF9 may constitute the predominant MET target. However, at MET concentrations (mM range) requisite to lower glucose concentrations from diabetic levels, MET effects may require the co-activation of both steroid receptor and the well-established MET metabolic (AMPK/mTOR) pathways. Further studies to address this hypothesis are ongoing in our laboratory.

Limitations of this study include the small patient sample size, the short duration of MET treatment, and the lack of pre-treatment patient samples (from initial biopsies) that would allow for a more accurate evaluation of MET effects. Moreover, our in vivo findings did not mimic the in vitro effects of MET on cell proliferation and apoptosis, as might be expected from their distinct contexts and treatment duration. Nevertheless, our findings provide support to the emerging body of evidence on the potential preventative use of MET for non-diabetic women who are at high risk for EC and offer mechanistic insights on steroid receptor signaling as a key pathway mediated by MET. The promising findings presented herein warrant further studies with a larger non-diabetic patient population to expand current understanding of novel networks to exploit for prevention of EC.

## Electronic Supplementary Material

ESM 1 (PDF 43 kb)

ESM 2 (DOCX 17 kb)

## Abbreviations

AMPK: 5′ Adenosine monophosphate-activated protein kinase
BMI: Body mass index
CCND1: Cyclin D1
CON: Control
DKK1: Dickkopf-related protein 1
EC: Endometrial carcinoma
ERα: Estrogen receptor-alpha
GE: Glandular epithelial
KLF4: Krüppel-like factor 4
KLF9: Krüppel-like factor 9
MAPK: Mitogen-activated protein kinase
MET: Metformin
MTOR: Mammalian target of rapamycin
PTEN: Phosphate and tensin homolog
PGR: Progesterone receptor
PGR-B: Progesterone receptor isoform B
siRNA: Small interfering RNA
ST: Stromal
TBP: TATA-binding protein
