# Supplementary material for: Metformin Promotes Anti-tumor Biomarkers in Human Endometrial Cancer Cells
Source: Reprod Sci. 2020 Jan 1;27(1):267–77. doi: 10.1007/s43032-019-00019-2 (PMC7077930; doi:10.1007/s43032-019-00019-2)
Supplement: Supplementary file 1 — (PDF 43 kb) [file 43032_2019_19_MOESM1_ESM.pdf]

Supplemental Table S1. Oligonucleotide primers used for quantitative RT-PCR

Gene Name	Forward Primer	Reverse Primer	PCR Product Size (bp)
CCND1	5'-CTG GCC ATG AAC TAC CTG-3'	5'-GTC ACA CTT GAT CAC TCT GG-3'	182
c-FOS	5'- TGT CTG TGG CTT CCC TTG ATC-3’	5'- TGG ATG ATG CTG GGA ACA GG-3’	189
DKK1	5’-GATCATAGCACCTTGGATGG-3'	5’-GATCATAGCACCTTGGATGG-3'	120
ER-α	5'-CGG CAT TCT ACA GGC CAA ATT-3'	5'-AGC GAG TCT CCT TGG CAG ATT-3'	120
KLF4	5'-TTCCCATCTCAAGGCACACCT-3'	5'-TGTTTACGGTAGTGCCTGGTCA-3'	111
KLF9	5'-TGG CTG TGG GAA AGT CTA TGG-3'	5'-CTC GTC TGA GCG GGA GAA CT-3'	124
PGR	5’-CCT TTGGAAGGGCTACGAAGT-3’	5’-GAGCTCGACACAACTCCTTTTTG-3’	110
PGR-B	5’-CGACCCAGGAGGTGGAGAT-3’	5’-GAGGGAAAAGGGAAGGAGGAG-3’	105
TP53	5'-GGC GCA CAG AGG AAG AGA AT-3'	5'-GGA GAG GAG CTG GTG TTG TTG-3'	103
PTEN	5'-GGC GGT GTC ATA ATG TCT TTC -3'	5'-GGC GGT GTC ATA ATG TCT TTC A-3'	138
TBP	5’-TCCACAGTGAATCTTGGTTGTA-3’	5’-CCTCATGATTACCGCAGCAAA-3’	102
TERT	5'-ATTCCTGCTCAAGCTGACTCGAC-3'	5'-ATGGTCTTGAAGTCTGAGGGCAG-3'	159
