# Supplementary material for: Metformin Promotes Anti-tumor Biomarkers in Human Endometrial Cancer Cells
Source: Reprod Sci. 2020 Jan 1;27(1):267–77. doi: 10.1007/s43032-019-00019-2 (PMC7077930; doi:10.1007/s43032-019-00019-2)
Supplement: Supplementary file 2 — (DOCX 17 kb) [file 43032_2019_19_MOESM2_ESM.docx]

**Supplemental Table S1.** Oligonucleotide primers used for quantitative RT-PCR

| Gene Name | Forward Primer | Reverse Primer | PCR Product Size (bp) |
| --- | --- | --- | --- |
| *CCND1*  *c-FOS*  *DKK1* | 5'-CTG GCC ATG AAC TAC CTG-3'  5'- TGT CTG TGG CTT CCC TTG ATC-3’  5’-GATCATAGCACCTTGGATGG-3' | 5'-GTC ACA CTT GAT CAC TCT GG-3'  5'- TGG ATG ATG CTG GGA ACA GG-3’  5’-GATCATAGCACCTTGGATGG-3' | 182  189  120 |
| *ER-α*  *KLF4*  *KLF9* | 5'-CGG CAT TCT ACA GGC CAA ATT-3'  5'-TTCCCATCTCAAGGCACACCT-3'  5'-TGG CTG TGG GAA AGT CTA TGG-3' | 5'-AGC GAG TCT CCT TGG CAG ATT-3'  5'-TGTTTACGGTAGTGCCTGGTCA-3'  5'-CTC GTC TGA GCG GGA GAA CT-3' | 120  111  124 |
| *PGR* | 5’-CCT TTGGAAGGGCTACGAAGT-3’ | 5’-GAGCTCGACACAACTCCTTTTTG-3’ | 110 |
| *PGR-B* | 5’-CGACCCAGGAGGTGGAGAT-3’ | 5’-GAGGGAAAAGGGAAGGAGGAG-3’ | 105 |
| *TP53*  *PTEN*  *TBP*  *TERT* | 5'-GGC GCA CAG AGG AAG AGA AT-3'  5'-GGC GGT GTC ATA ATG TCT TTC -3'  5’-TCCACAGTGAATCTTGGTTGTA-3’  5'-ATTCCTGCTCAAGCTGACTCGAC-3' | 5'-GGA GAG GAG CTG GTG TTG TTG-3'  5'-GGC GGT GTC ATA ATG TCT TTC A-3'  5’-CCTCATGATTACCGCAGCAAA-3’  5'-ATGGTCTTGAAGTCTGAGGGCAG-3' | 103  138  102  159 |
